# Postnatal Changes in Tibial Bone Speed of Sound of Preterm and Term Infants during Infancy

**DOI:** 10.1371/journal.pone.0166434

**Published:** 2016-11-10

**Authors:** Hsiu-Lin Chen, Wei-Te Lee, Pei-Lun Lee, Po-Len Liu, Rei-Cheng Yang

**Affiliations:** 1 Department of Pediatrics, Kaohsiung Medical University Hospital, Kaohsiung, Taiwan; 2 Department of Respiratory Therapy, College of Medicine, Kaohsiung Medical University, Kaohsiung, Taiwan; 3 School of Medicine, College of Medicine, Kaohsiung Medical University, Kaohsiung, Taiwan; Nanjing Medical University, CHINA

## Abstract

This study aimed to evaluate changes in tibial bone speed of sound (SOS) over time, in preterm and term infants during infancy, in addition to identifying factors influencing the development of tibial SOS during infancy. Preterm (n = 155) and term (n = 65) infants were enrolled in this study. Tibial bone SOS was measured using quantitative ultrasonography (QUS) on the left tibia of newborn infants after birth (within 7 days), at 1 month old, and then every 2 months until subjects were approximately 12–15 months old. Follow-up checks included anthropometric measurements and tibial bone SOS. Mean tibial bone SOS at birth was significantly higher in term infants (mean ± SD, 2968.5 ± 99.7 m/s) than in preterm infants (2912.2 ± 122.6 m/s). Values of follow-up tibial bone SOS declined for the first 4 months, and then increased gradually until 12–15 months old. This increasing trend was greater in preterm infants after 2 months of corrected age than in term infants. There were no significant differences by 12–15 months of age between preterm and term infants. A longitudinal mixed-effect model controlling for internal correlations and other covariates in the two groups showed that age and the SOS value at birth were important factors affecting the tibial bone SOS in both preterm and term newborn infants during infancy. There are significant differences in the pattern of change in tibial bone SOS values between preterm and term infants during the first 12–15 months of life. Age and SOS value at birth were important factors affecting the pattern of tibial bone SOS change in both preterm and term newborn infants during infancy.

## Introduction

Given that the rates of osteoporosis is reportedly increasing in all demographic groups [1, 2], it is important to understand factors associated with bone strength and development in newborn infants, as a means of creating preventive strategies against osteoporotic fracture for future generations. Epidemiological studies have demonstrated a relationship between birth weight, weight in infancy, and adult bone mass [3, 4]. Hence, fetal and neonatal periods are critical to bone growth and may correlate with skeletal strength in later life. Preterm infants have a particularly high risk for osteopenia of prematurity, also known as bone metabolic disorder of prematurity [5, 6]. Whether a different pattern of change in bone status between preterm and term infants exists in later life remains to be identified.

Measuring bone development precisely in newborns and infants is a methodological challenge. Dual-energy X-ray absorptiometry (DEXA) is now well defined as an assessment method for the accretion of minerals in adults, and is the current standard for whole-body measurement of minerals [7]. However, DEXA is not suitable for the assessment of neonatal bone status because it requires specialized instruments, experienced personnel, prolonged examination time, and exposure to radiation [8, 9]. Quantitative ultrasound (QUS) displays the ultrasound waveform and automatically detects the fastest waves transmitted through the bone [10, 11], and can be used as a relatively inexpensive, portable, noninvasive, and radiation-free method of assessing bone status. Bone status at different skeletal sites has been investigated. The report by McDevitt et al. [12] showed the variability in reproduction of SOS at radial and tibial sites. Koo et al. [8] demonstrated that SOS is much more difficult to obtain at the radius than at tibia in infants. Hence, bone SOS values were measured on tibial bone in the majority of studies with infants. Several studies have reported on the application of QUS in assessing the bone status in newborn preterm infants [13] and osteopenia of prematurity [10, 14] and following skeletal development and maturation by QUS in preterm infants [15].

Although there have been a number of reports on QUS-related assessment of bone status in children and adolescents [16–18], few studies have assessed postnatal bone status during infancy. The aims of this study were to evaluate postnatal changes in tibial bone SOS over time in preterm and term infants during the first year of life, and to determine what factors influence development of tibial bone SOS during infancy.

## Materials and Methods

### Subjects

Term (gestational age [GA] ≥ 37 weeks) and preterm (GA < 37 weeks) infants admitted to the baby room or neonatal ward of Kaohsiung Medical University Hospital between October 2007 and July 2015 were enrolled in this study. Infants with major congenital anomalies or congenital chromosomal disorders were excluded. Following enrollment, a birth chart including gestational age, birth body weight, and birth height was recorded. Infants with weight below the 10th percentile for GA (small for GA) and above the 90th percentile for GA (large for GA), based on intrauterine growth charts in Taiwan [19], and multiple births were excluded from this study. The ponderal index (PI) was calculated as weight in kilograms divided by the body length in meters cubed (kg/m^3^) [20] to approach body composition in study infants.

### QUS measurement of the tibial bone SOS

QUS measurement of the tibial bone SOS was performed using an appropriate sized probe with the Sunlight Omnisense 7000S Quantitative Ultrasound scanner (Sunlight Medical Ltd., Israel), which is based on the technique of ultrasound critical angle reflectometry, according to the manufacturer’s instructions. There are three different size probes with a frequency of 1.25 MHz (CS—contact surface 2.5 × 1 cm, CR—contact surface 3.1 × 1.2 cm, CM—contact surface 4.2 × 1.3 cm), chosen appropriately for infants of different body weight. The CS probe is used for infants weighing less than 3 kg, the CR probe for infants weighing 3–5 kg, and the CM probe for infants weighing more than 5 kg. Infant weight is used when choosing the probe because the thickness of soft tissue can result in unsuccessful measurements (lack of recording of SOS during a measurement cycle) if the probe used is inappropriately sized for infant weight. The same technician performed all measurements. System quality verification of the Sunlight Omnisense 7000S Quantitative Ultrasound scanner was performed each day using a phantom, provided by the manufacturer, and the coefficients of variation for the CS, CR, and CM probes were 0.24%, 0.42%, and 0.31%, respectively. Five neonates weighing less than 3 kg (CS probe), 5 infants weighing 3–5 kg (CR probe), and 5 infants weighing more than 5 kg (CM probe) were measured 5 times each to determine the precision of each probe. Coefficient variation for the CS, CR, and CM probes were 0.92%, 1.31%, and 1.31%, respectively. In this longitudinal study, all enrolled infants were measured with at least 2 different probes and, and in most cases, with all 3 probes. In order to validate the consistency of SOS measurements using 3 different probes, paired SOS measurements were performed by using CS and CR probes in 7 infants weighing 1.5–3.1 kg, and CR and CM probes in 7 infants weighing 3.5–4.1 kg. There was strong correlation between CS and CR probes (r = 0.92, p<0.0001), and between CR and CM probes (r = 0.78, p = 0.0004).

Tibial bone SOS was measured at the middle of the left tibia. The first measurement was performed within seven days after birth (range 0–7 days, median 3 days). Infants returned to the hospital after discharge for a routine follow-up at 1 month old, and then every 2 months until they were 12–15 months old. Follow-up checks included measurements of body weight, height, and tibial bone SOS. For preterm infants, corrected age (CA) is calculated as postnatal age (PNA) reduced by the number of weeks born before 40 weeks of gestation [21].

### Ethics statement

This study was approved by the Institutional Review Board (IRB) of Kaohsiung Medical University Hospital (number KMUH-IRB-980555). Informed consent was documented by the use of a written consent form approved by the IRB and signed by the parents of all infants prior to enrollment. The investigator gave the parents adequate opportunity to read it before it was signed.

### Statistical analysis

Continuous variables are presented as mean and standard deviation (SD), and categorical variables as number and percentage. Chi-square tests and t-tests were used to compare basic characteristics among groups. Polynomial regression analysis was used to identify the best fitting model (less than 1% increase in r^2^) that was able to reflect the pattern of change in bone SOS with age. Univariate analysis was used to identify possible factors influencing values of tibial bone SOS in both groups. A longitudinal mixed-effect model was further used to test for associations between bone SOS and possible influencing factors, an analytic approach that permits full use of available data while controlling for internal correlations and other covariates in two groups of infants. This model treats each bone SOS data from each enrolled infants as a separate observation and adjusts for within-infant correlations and correlations with their prior bone SOS data. Subjects were treated as random effects, and the analysis could be adjusted to each infant’s bone SOS data. A first-order autoregressive error structure accounted for within-infant correlation. A 2-tailed p value less than 0.05 was considered statistically significant. All statistical analyses were performed using the SAS® statistical software version 9.3 (SAS Institute Inc., Cary, NC).

## Results

A total of 220 infants, including 65 term and 155 preterm infants, were enrolled between October 2007 and July 2015. There were 37 (23.9%) preterm infants with birth weight less than 1000 g, whose corresponding gestation was 24–28 weeks. Sixty-two (40.0%) preterm infants weighed 1000–1499 g at birth, with corresponding gestation of 26–31 weeks. The remaining 56 (36.1%) preterm infants weighed more than 1500 g at birth with corresponding gestation of 29–36 weeks. The QUS procedure was well tolerated in all infants during the study period, and no adverse effects were observed. There was a total of 1352 tibial bone SOS and anthropometric measurements taken from enrolled infants between birth and 12–15 months old.

Clinical characteristics of infants enrolled in the study are shown in Table 1. In addition to significant differences in GA and anthropometric measurements between preterm and term infants, there was also a significantly higher percentage of maternal hypertension during pregnancy and history of premature rupture of membrane (PROM) in preterm infants. Mean tibial bone SOS at birth was significantly higher in term infants (2968.5 ± 99.7 m/s) than in preterm infants (2912.2 ± 122.6 m/s).

**Table 1:** Clinical characteristics of study infants.

	Preterm babies (< 37 wks) N = 155	Term babies (≥ 37 wks) N = 65	p value
**Gestational age, weeks**	29.1 ± 2.8	38.6 ± 0.8	< 0.0001
**Birth weight, g**	1407.6 ± 516.9	3180.5 ± 360.3	< 0.0001
**Birth height, cm**	39.1 ± 4.8	50.5 ± 2.3	< 0.0001
**Birth head circumference, cm**	27.0 ± 3.1	33.4 ± 1.4	< 0.0001
**Body mass index at birth**	8.9 ± 1.7	12.5 ± 1.4	< 0.0001
**Ponderal index**	2.3 ± 0.4	2.5 ± 0.3	< 0.0001
**Male sex, n (%)**	84 (54.2)	32 (49.2)	0.501
**Maternal age, y**	31.5 ± 5.7	31.2 ± 3.4	0.645
**Vaginal delivery, n (%)**	86 (55.5)	37 (56.9)	0.845
**Primiparous, n (%)**	61 (39.4)	28 (43.1)	0.608
**History of PROM, n (%)**	34 (22.2)	2 (3.1)	< 0.001
**Hypertension during pregnancy, n (%)**	22 (14.4)	1 (1.5)	< 0.001
**Diabetes mellitus, n (%)**	7 (4.6)	1 (1.5)	0.275
**Maternal smoking, n (%)**	3 (2.0)	1 (1.5)	0.836
**Maternal alcohol, n (%)**	2 (1.3)	0 (0)	0.356
**Season of birth, n (%)**			0.211
**Spring**	35 (22.6)	21 (32.2)	
**Summer**	39 (25.2)	12 (18.5)	
**Autumn**	42 (27.0)	12 (18.5)	
**Winter**	39 (25.2)	20 (30.8)	
**Tibial bone SOS, m/sec**	2912.2 ± 122.6	2968.5 ± 99.7	< 0.001

Continuous data are presented as mean ± standard deviation (SD). SOS, speed of sound; PROM, premature rupture of membranes.

There was no mortality among 155 preterm infants. Mean length of hospitalization was 69.6 ± 37.8 days for preterm infants. Fifty-five preterm infants (35%) were diagnosed as having bronchopulmonary dysplasia, which was defined as oxygen dependency at postmenstrual age 36 weeks. Seventy-three preterm infants (47.1%) were prescribed diuretics (furosemide/thiazides) more than 7 days. The body weight, body length and bone SOS in preterm infants during hospitalization were 2.1 ± 0.9 kg, 42.3 ± 5.6 cm, and 2811.8 ± 113.6 m/s at the first month, 2.5 ± 0.9 kg, 43.6 ± 5.1 cm, and 2759.0 ± 119.8 m/s at the second month, and 3.7 ± 0.8 kg, 48.5 ± 5.4 cm, and 2747.3 ± 308.0 m/s at the third month, respectively.

Table 2 shows the number of different size probes used in this study according to age group. An appropriate size probe was chosen for infants of different body weight. When infants grew into a larger weight category, they needed larger size probe, i.e. the CM probe. One thousand and two (74.1%) tibial bone SOS measurement were collected with the CM probe, whereas 12.8% and 13.1% of measurements were collected with the CS and CR probes, respectively.

**Table 2:** The number of tibial bone SOS measurements with different size probes by age.

	CS probe	CR probe	CM probe
**#CGA<40w**	162	54	14
**0-1m**	10	48	16
**1-2m**	5	51	76
**2-4m**	0	20	185
**4-6m**	0	0	192
**6-8m**	0	0	162
**8-10m**	0	0	132
**10-12m**	0	0	137
**12-15m**	0	0	88
**Total (%)**	177 (13.1)	173 (12.8)	1002 (74.1)

#CGA: corrected gestational age.

Table 3 shows the tibial bone SOS and growth variables for term and preterm infants during the study period by age class (preterm infants by CA) and sex. There was no significant difference in tibial bone SOS between sexes, excluding that observed in the age class of 6–8 months and 12–15 months of corrected age in preterm infants when mean SOS values were higher in girls.

**Table 3:** Tibial bone SOS and growth variables for term and preterm infants during the study period by age and sex.

Group	Age (months)	n	SOS (m/s)	Height (cm)	Weight (kg)	PI (kg/m^3^)
**Term Boys**	**Birth**	32	2967.7 ± 94.8	51.0 ± 2.0	3.3 ± 0.3	24.7 ± 2.8
	**1-2m**	11	2820.9 ± 94.0	55.5 ± 2.4	4.8 ± 0.5	28.4 ± 2.4
	**2-4m**	12	2819.0 ± 110.8	58.8 ± 2.3	6.0 ± 0.7	29.6 ± 2.8
	**4-6m**	20	2872.4 ± 69.0	64.5 ± 2.2	7.6 ± 0.9	28.6 ± 3.8
	**6-8m**	22	2985.3 ± 94.6	68.2 ± 2.3	8.4 ± 1.0	26.3 ± 2.5
	**8-10m**	11	3062.6 ± 73.1	70.7 ± 3.0	9.2 ± 1.2	25.9 ± 2.5
	**10-12m**	17	3083.7 ± 91.0	73.4 ± 2.1	9.5 ± 1.0	24.1 ± 2.1
	**12-15m**	26	3160.2 ± 101.0	76.9 ± 3.0	10.2 ± 1.0	22.4 ± 1.0
**Term Girls**	**Birth**	33	2969.7 ± 105.7	50.1 ± 2.5	3.1 ± 0.4	24.8 ± 3.7
	**1-2m**	16	2840.8 ± 124.8	54.4 ± 3.0	4.5 ± 0.7	27.9 ± 3.3
	**2-4m**	12	2877.3 ± 81.9	58.0 ± 2.1	5.4 ± 0.9	27.5 ± 3.0
	**4-6m**	18	2898.0 ± 101.3	63.4 ± 2.5	7.2 ± 0.8	28.1 ± 2.9
	**6-8m**	17	2974.3 ± 101.7	68.0 ± 2.3	8.1 ± 1.0	25.7 ± 2.7
	**8-10m**	19	3023.7 ± 95.5	70.1 ± 2.9	8.6 ± 1.2	25.1 ± 2.8
	**10-12m**	19	3093.1 ± 62.1	73.6 ± 3.2	9.2 ± 1.2	23.1 ± 2.7
	**12-15m**	28	3185.3 ± 78.7	75.7 ± 2.4	9.7 ± 1.2	22.4 ± 2.2
**#Preterm Boys**	**Birth**	84	2914.2 ± 116.5	39.2 ± 4.9	1.4 ± 0.6	23.3 ± 4.2
	**1-2m**	55	2765.2 ± 123.3	55.5 ± 3.3	5.1 ± 1.0	29.5 ± 3.3
	**2-4m**	96	2837.5 ± 123.1	60.3 ± 3.9	6.2 ± 1.2	28.2 ± 3.3
	**4-6m**	84	2938.1 ± 120.2	64.5 ± 3.5	7.4 ± 1.3	27.4 ± 3.0
	**6-8m**	66	2997.2 ± 105.2	67.3 ± 3.4	7.9 ± 1.4	25.6 ± 2.8
	**8-10m**	51	3083.2 ± 95.7	70.3 ± 3.4	8.4 ± 1.5	24.1 ± 2.3
	**10-12m**	51	3177.1 ± 81.4	72.6 ± 3.4	8.7 ± 1.4	22.8 ± 2.4
	**12-15m**	17	3177.8 ± 72.0	75.4 ± 4.4	9.9 ± 1.4	23.0 ± 1.9
**#Preterm Girls**	**Birth**	71	2910.0 ± 130.3	39.0 ± 4.7	1.4 ± 0.5	22.7 ± 4.8
	**1-2m**	50	2714.9 ± 149.8	55.1 ± 3.9	48.0 ± 0.9	28.6 ± 4.2
	**2-4m**	85	2813.7 ± 126.4	60.0 ± 3.6	6.0 ± 1.0	27.6 ± 3.2
	**4-6m**	70	2944.9 ± 107.9	64.5 ± 3.2	7.1 ± 1.0	26.3 ± 3.1
	**6-8m**	57	3042.2 ± 97.1	68.3 ± 3.1	7.8 ± 1.0	24.6 ± 2.9
	**8-10m**	51	3111.7 ± 80.7	71.2 ± 3.4	8.6 ± 1.1	23.7 ± 2.5
	**10-12m**	50	3180.2 ± 73.6	74.0 ± 3.1	8.8 ± 1.2	21.8 ± 2.0
	**12-15m**	17	3238.8 ± 97.0	75.0 ± 3.7	9.3 ± 1.2	22.1 ± 2.5

Continuous data are presented as mean ± SD. SOS, speed of sound; PI, ponderal index.

#: Age groups in preterm infants are based on corrected age in months.

**Fig 1:**
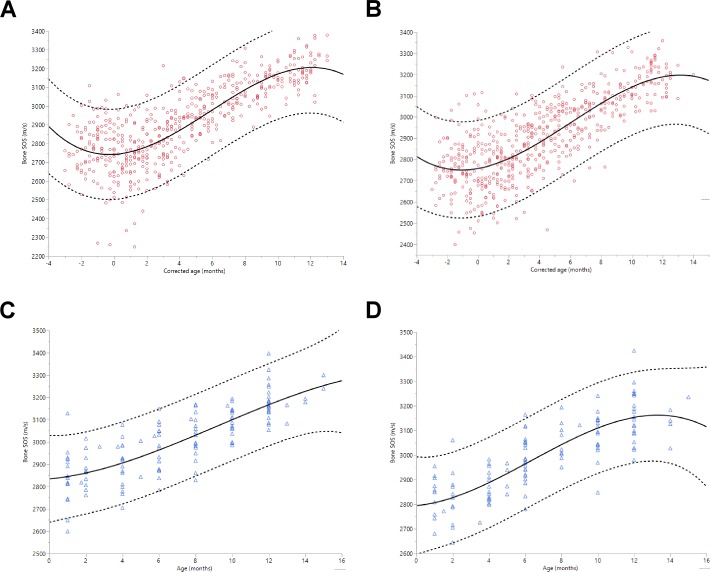
Scatter plots of tibial bone SOS in preterm and term infants with a cubic regression line versus age in months. (A) preterm girls, (B) preterm boys, (C) term girls, (D) term boys. Corrected age is used for preterm infants. Upper and lower individual 95% confidence intervals are shown as dashed lines. ○ = preterm infants; △ = term infants; values on X axis of Fig 1 (A) and (B): zero value = corrected gestational age 40 weeks; negative values -1 = corrected gestational age 36 weeks, negative -2 = corrected gestational age 32 weeks, negative -3 = corrected gestational age 28 weeks.

The relationship of bone SOS with age in months was evaluated by polynomial regression analysis for curve fitting. The best fitting model was a third degree polynomial in both groups by sex. Fig 1A–1D show the bone SOS values as a function of age and the fitted regression curves for girls and boys of term and preterm infants separately. In preterm infants, both sexes revealed the nadir of bone SOS at a corrected age of 0–2 months. Thereafter, there is an increase and a plateau reached up to age 10–12 months. In term infants, tibial bone SOS positively correlated with age in months in both sexes, although a slight decreasing trend of bone SOS between birth and 2 months of age was noted. For term boys, there was a slightly decreasing trend after 12 months of age.

**Fig 2:**
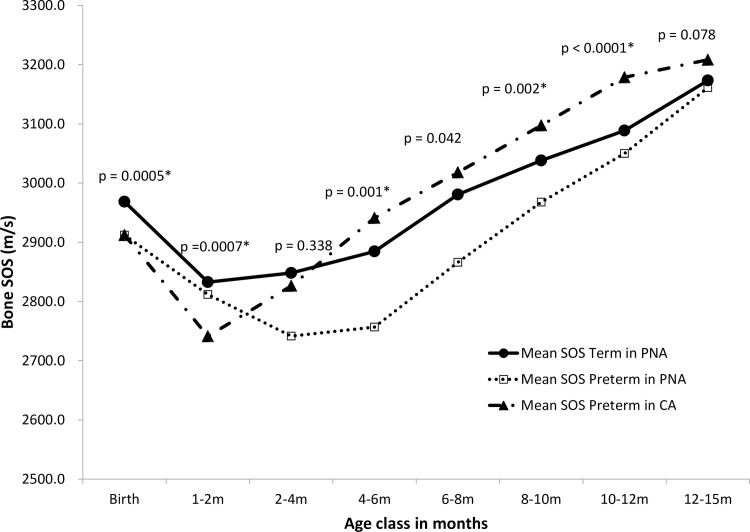
Trends in age-related changes for mean tibial bone SOS in terms of postnatal age (PNA) and preterm infants of PNA and corrected age (CA) during infancy. Tibial bone SOS in each age class of PNA in term infants and CA in preterm infants was compared using t tests. The corresponding p value (PNA in term infants versus CA in preterm infants) is shown for each age class. Post-hoc Bonferroni adjustment was applied to p values to account for multiple comparisons; hence, p values < 0.006 (≈ 0.05/8) were considered statistically significant (*) between term infants in PNA and preterm infants in CA.

Trends in age-related changes for tibial bone SOS in term and preterm infants are shown in Fig 2. Three lines with mean bone SOS value versus age class are plotted to show changes in the trend between PNA in term and preterm infants, and CA in preterm infants. Higher tibial bone SOS values were observed in the term group after birth, when compared to the preterm group. There was a decreasing trend for tibial bone SOS values in both groups. The nadir point occurred at CA 1–2 months of age in the preterm groups, significantly lower than that of the term groups. As shown in Fig 2, the decreasing trend in tibial bone SOS values during the first 1 to 2 months of CA in preterm infants was greater than that of term infants. However, in contrast, preterm infants exhibited a steeper increase in tibial bone SOS values after 2 months of CA; thus, the values of tibial bone SOS of preterm infants were significantly higher than that of term infants at age classes of CA 4–6, 8–10, and 10–12 months of age. By 12–15 months old, there was no significant difference between the groups.

Univariate analysis to identify possible factors influencing values of tibial bone SOS in both groups revealed that advanced age class (term infants in PNA and preterm infants in CA), body weight, height at the measured time, and SOS at birth positively affected tibial bone SOS development after birth, whereas sex and maternal smoking during pregnancy did not. PI and status of breastfeeding at the measured time were negatively associated with tibial bone SOS in both preterm and term infants. In preterm infants, GA, birth body weight and birth body height, but not PI at birth, were also significant factors influencing development of tibial bone SOS during infancy. In term infants, PI at birth was a negative factor for development of tibial bone SOS.

We further preceded multivariate analysis by using a longitudinal mixed-effect model to test for associations between bone SOS and possible influencing factors. Collinearity may occur among body weight, body height, and PI (also between birth body weight, birth body height, and PI at birth) in regression; therefore, we selected PI as an anthropometric status measure for the multivariate analysis. The variables of age class, sex, GA, SOS value at birth, anthropometric status at birth, season of birth, status of breastfeeding at the measured time, and PI at the measured time were included in the multivariate analysis for both preterm and term infants. The results revealed that age and the SOS value at birth remained significant positive factors influencing tibial bone SOS development during infancy for term infants. For preterm infants, PI at the measured time was an important factor with a negative effect on increased tibial bone SOS during infancy. Status of breastfeeding at the measured time and season of birth in multivariate analysis did not show significant effects on tibial bone SOS during infancy in both groups.

## Discussion

The present study provides important information on the postnatal changes in tibial bone SOS for preterm and term infants from birth to 12–15 months of age. There are significant differences in the pattern of change in tibial bone SOS values between preterm and term infants during infancy. Furthermore, we showed that the most important factors associated with bone strength as a reflection of tibial bone SOS in both preterm and term infants were age in months, and the tibial bone SOS value at birth.

Prevrhal et al. have shown that the tibial bone SOS measurement depends on both the thickness and density of the tibia, and is influenced by the density of the cortex near the surface, rather than the internal structure [22]. Given this relationship with surrogate markers for bone strength, and the safety and convenience of measuring tibial bone SOS, this may be a feasible approach for assessing bone status in infants.

The differences in factors influencing tibial bone SOS between preterm and term infants may be explained by maturity of the fetus. The majority of calcium accretion occurs during the third trimester, with peak accretion rates between 36 and 38 weeks of gestation [4, 23]. When babies are born at term, it may be assumed that a satisfactory amount of bone accretion and nutrition has been acquired, and thus we would expect better tibial bone SOS and anthropometric measurements at birth. In contrast to term infants, preterm infants are born early, and do not achieve adequate bone accretion and nutritional status. Hence, advanced gestational age and greater anthropometric measurements at birth are reflective of satisfactory bone accretion and nutrition, which may positively influence bone SOS value at birth and further influence bone growth in later life.

The present study revealed that there were no sex-based differences in results of both univariate and multivariate analysis. In our previous study on bone status at birth [24], we showed that tibial bone SOS was higher in male than female infants for an early GA time point; however, at late GA, male and female infants were closer to the linear regression. This phenomenon might explain why previous reports revealed no difference in sex at term birth [13, 25–27]. After birth, both sexes exhibit the same pattern of development of bone SOS during infancy in our study. Koo et al. also reported that gender failed to show a statistical significant effect on postnatal development of bone mineral status as measured by dual energy X ray absorptiometry [28].

Interestingly, we found a decreasing trend in tibial bone SOS for both groups after birth, until 4 months old, which was more evident in preterm infants. After this period of decrease, tibial bone SOS values were observed to increase gradually until 1 year of life. The same phenomenon has been reported in Caucasian [15] and Chinese populations [26]. At birth, about 92% of the cross-section is cortical bone. As the bone-marrow cavity increases during the first months of life, the bone mineral density of long bones drops by about 30%, and cortical thickness decreases [3, 29]. This phenomenon may explain the decreasing trend in tibial bone SOS values from both groups in the first 5 months after birth. After that, tibial bone SOS values increased until 1 year old in both groups. Preterm infants exhibited a faster increase than full-term infants after 2 months of CA. By 12–15 months old, there was no significant difference between the groups. This indicates that, although there are significantly lower tibial bone SOS values at birth in preterm infants, catch-up growth occurs to narrow this difference.

In our study, we used PI to approach body mass. Although PI and body mass index are poor predictors of adiposity at birth [20], PI is still suggested as a complementary measurement in neonatal and pediatric growth and nutritional assessment. In preterm infants, PI at measured time showed a negative effect on development of bone SOS, but this effect was not seen in term infants. Higher PI suggests increased adiposity in infants. Whether increased adiposity (obesity) affects the development of bone SOS in later life requires further clarification from long-term follow-up studies.

The limitation of our study is that we did not account for other potential confounders, such as complementary feeding, sunlight exposure, activity and hormonal status of the enrolled infants. Moreover, subcutaneous fat and probe size can confound the QUS measurements. Tibial bone measurement by the same probe cannot be used due to growth status during a longitudinal study. Infant size appears to significantly affect bone SOS measurements at the tibia, with the smaller probe tending to be unsuccessful in infants with larger weights. Hence, validity of QUS data include SOS as surrogate for bone status remains to be defined.

The strength of this study is that we used a longitudinal mixed-effect model analysis with a first-order autoregressive error structure to test for associations between bone SOS and possible influencing factors while accounting for within-infant correlation. Even though there were limitations to this study, our results provide important information on tibial bone SOS values, according to age in months and sex during infancy, for both term and preterm infants. We also observed differences in the pattern of postnatal tibial bone SOS changes between groups. Given the limited data in the literature pertaining to these trends, this study could be useful for evaluating bone status in both preterm and term infants during their infancy.

## Conclusion

This study provided information of postnatal changes in tibial bone SOS for preterm and term infants during infancy. There are significant differences in the pattern of change in tibial bone SOS values between preterm and term infants during the first year of life. For both preterm and term infants, age and the SOS value at birth are important factors affecting the pattern of tibial bone SOS change during infancy.
